# Dependable and Efficient Clinical Molecular Diagnosis of Chinese RP Patient with Targeted Exon Sequencing

**DOI:** 10.1371/journal.pone.0140684

**Published:** 2015-10-23

**Authors:** Liping Yang, Hui Cui, Xiaobei Yin, Hongliang Dou, Lin Zhao, Ningning Chen, Jinlu Zhang, Huirong Zhang, Genlin Li, Zhizhong Ma

**Affiliations:** 1 Department of Ophthalmology, Peking University Third Hospital, Key Laboratory of Vision Loss and Restoration, Ministry of Education, Beijing, P. R. China; 2 Beijing Tongren Eye Center, Beijing Tongren Hospital, Capital Medical University, Beijing, P. R. China; Hadassah-Hebrew University Medical Center, ISRAEL

## Abstract

Retinitis pigmentosa (RP) is the most common inherited retinal disease. It is a clinically and genetically heterogeneous disorder, which is why it is particularly challenging to diagnose. The aim of this study was to establish a targeted next-generation sequencing (NGS) approach for the comprehensive, rapid, and cost-effective clinical molecular diagnosis of RP. A specific hereditary eye disease enrichment panel (HEDEP) based on exome capture technology was used to collect the protein coding regions of 371 targeted hereditary eye disease genes, followed by high-throughput sequencing on the Illumina HiSeq2000 platform. From a cohort of 34 Chinese RP families, 13 families were successfully diagnosed; thus, the method achieves a diagnostic rate of approximately 40%. Of 16 pathogenic mutations identified, 11 were novel. Our study demonstrates that targeted capture sequencing offers a rapid and effective method for the molecular diagnosis of RP, which helps to provide a more accurate clinical diagnosis and paves the way for genetic counseling, family planning, and future gene-targeted treatment.

## Introduction

Retinitis pigmentosa (RP) is the most frequent subtype of inherited retinal disease and it is a clinically and genetically highly heterogeneous disorder [1]. RP affects approximately 1 in 3,500 individuals worldwide [2], and data from the Beijing Eye Institute suggest that its prevalence may be even higher in China (approximately 1 in 1,000) [3]. Typical symptoms of RP include night blindness, followed by decreasing visual fields leading to tunnel vision, and eventually blindness. Clinical hallmarks include bone-spicule deposits, attenuated retinal blood vessels, optic disc pallor, visual field loss, and abnormal, diminished, or non-recordable electroretinographic responses. To date, 74 genes have been associated with non-syndromic RP (RetNet; http://www.sph.uth.tmc.edu/Retnet/), which makes the dependable and efficient clinical molecular diagnosis of RP patients particularly challenging.

The identification of a causative mutation is important to ascertain the genetic basis of the disease, which in turn paves the way for genetic counseling, family planning, and future gene-targeted treatment [4]. PCR-based Sanger sequencing is the gold standard for clinical molecular diagnosis; however, with a few exceptions, there are no ophthalmologic characteristics specifically associated with the genetic subtypes of RP, impeding the prioritization of genes for analysis by Sanger sequencing [5]. Widely used microarray-based genotyping technologies, such as the GoldenGate assay with VeraCode microbeads [6] and the arrayed primer extension (APEX) chip [7], enable the simultaneous detection of multiple mutations from one individual. However, they are limited by the fact that they only detect known mutations, while novel mutations elsewhere in a known gene go unreported. Thus, the positive diagnostic rates of these methods are low. Next-generation sequencing (NGS) enables rapid and cost-effective parallel sequencing of a large panel of disease genes, and provides a promising alternative for the molecular diagnosis of RP [8]. Our previous studies demonstrate that a specific hereditary eye disease enrichment panel (HEDEP) based on exome capture technology can be of diagnostic value [9–10] and can have a practical application in clinical work.

This article describes the use of HEDEP in the clinical molecular diagnosis of 34 Chinese RP families and characterizes the phenotypic manifestation associated with the identified mutations.

## Materials and Methods

### Ethics Statement

The study conformed to the tenets of the Declaration of Helsinki. All experiments involving DNA and RNA of patients and their relatives were approved by the Medical Ethics Committee at the Peking University Third Hospital (No. 2012093). Written informed consent was obtained from all participants, or guardians on behalf of the minors. The ethics committee approved this consent procedure.

### Patients

A cohort of 34 Chinese RP families of Han ethnicity was recruited from the Department of Ophthalmology, Peking University Third Hospital, and Beijing Tongren Eye Center. The diagnosis of RP was based on night blindness, progressive loss of peripheral vision, decreasing visual acuity with age, waxy pale discs, retinal arteriolar attenuation, scattered bone-spicule pigmentation in the mid-peripheral retina, and reduced rod and cone function. Medical and ophthalmic histories were obtained, and ophthalmological examinations were carried out. There was no history of other ocular or systemic abnormalities in these families. One hundred healthy individuals from the Chinese Han ethnic population were recruited as controls.

### Mutation Screening with HEDEP

Blood samples were collected and genomic DNA was extracted by standard protocols (D2492 Blood DNA Maxi Kit, Omega Bio-Tek Inc., GA, USA). The HEDEP and high-throughput DNA sequencing were applied for the mutation screening. The HEDEP was able to capture 371 hereditary eye disease genes, covering 24 autosomal dominant RP (adRP)-associated genes (*BEST1*, *C1QTNF5*, *CA4*, *CRX*, *FSCN2*, *GUCA1B*, *IMPDH1*, *KLHL7*, *NR2E3*, *NRL*, *PRPF3*, *PRPF31*, *PRPF6*, *PRPF8*, *RDS*, *RDH12*, *RHO*, *ROM1*, *RP1*, *RP9*, *RPE65*, *SEMA4A*, *SNRP200*, and *TOPORS*) and 35 autosomal recessive RP (arRP)-associated genes (*ABCA4*, *BEST1*, *C2orf71*, *C8ORF37*, *CERKL*, *CLRN1*, *CNGA1*, *CNGB1*, *CRB1*, *DHDDS*, *EYS*, *FAM161A*, *IDH3B*, *IMPG2*, *LRAT*, *MAK*, *MERTK*, *NR2E3*, *NRL*, *PDE6A*, *PDE6B*, *PDE6G*, *PRCD*, *PROM1*, *RGR*, *RHO*, *RLBP1*, *RP1*, *RPE65*, *SAG*, *SPATA7*, *TTC8*, *TULP1*, *USH2A*, and *ZNF513*) that had been reported at the time of the panel design, including several overlapping genes. Targeted gene enrichment, high-throughput sequencing, and data analysis were done as described previously [10]. In brief, 50 μg of genomic DNA from 3 members of each family was used for targeted exome capture. The exon-enriched DNA libraries were then prepared for high-throughput sequencing on the Illumina HiSeq 2000 platform. The obtained mean exome coverage was more than 98%, with a variant calling accuracy of more than 99%. The changes were filtered against exome data from ethnic Han Chinese individuals from Beijing available in the 1000 Genomes Project (http://www.1000genome.org), and against the Han Chinese Beijing SNPs in the dbSNP131. The changes shared by the affected individuals but not the healthy controls were identified.

### Mutation Validation

The variants were confirmed by Sanger sequencing on an ABI 3130xl Genetic Analyzer (Applied Biosystems, Foster City, CA, USA). Sanger sequencing was also used to determine whether the variant co-segregated with the disease phenotype in these families and in 100 healthy controls. The possible pathogenicity of missense changes was further evaluated using SIFT, PolyPhen-2, and Mutation Taster prediction software and via evolutionary conservation analysis. Primer pairs were designed using the Yeast genome primer program (http://www.yeastgenome.org/cgi-bin/web-primer). Splice-site variants were analyzed using the prediction program AUGUSTUS (http://bioinf.uni-greifswald.de/augustus/submission) and Automated Splice Site Analyses (http://splice.uwo.ca/).

### Isolation of total RNA and reverse transcription (RT)-PCR analysis

To determine whether intron mutations had any effect on mRNA splicing, RT-PCR analysis was performed. Total RNA was extracted from peripheral whole blood samples by standard protocols (R6814 Blood RNA Kit, Omega) and reverse transcribed with oligonucleotide primers using Superscript II reverse transcriptase (Invitrogen Corporation, Grand Island, NY). Primers for RT-PCR were designed to amplify exon 7–12 of *PRPF31* mRNA (mRNA reference number NM_015629): forward primer 5′-GCCAAGATCATGGGTGTGG-3′, and reverse primer 5′-TGCAGCGTCTTGGAGATCCT-3′.

## Results

### Patients

In total, 34 Chinese RP families (17 adRP and 17 arRP families) were recruited in this study. All families had two or more affected patients, and their initial symptoms were night blindness followed by progressive loss of peripheral vision. The clinical features of the probands varied greatly: onset of the disease ranged from 3 to 28 years of age, and visual acuity ranged from light perception (LP) to 0.8. The patients also showed different degrees of retinal pigment epithelium (RPE) atrophy, retinal vascular attenuation, and bone spicule deposits.

### Targeted Sequencing and Bioinformatic Analysis

We selected three members of each family for targeted exon capture (with two patients and one control for adRP families; one patient and their parents for arRP families, if one of the parents had passed away, we chose another patient instead). High quality sequencing results were obtained. We generated at least 0.77 Gb of sequence with 228× average coverage for each individual with paired 100-bp reads. The generated sequence covered an average of 99.2% of the targeted bases with a variant calling accuracy of more than 99%, which is sufficient to pass the thresholds for calling SNPs and short insertions or deletions (indels). For every adRP family we filtered all the detected variants in the two patients against each other, since the two patients are related and are expected to share the same causal variant, and identified the shared variants. For every arRP family, a homozygous or compound heterozygous mutation of bi-parental inheritance was postulated to be the disease causing mutation. We filtered all the detected variants in the patient against his or her parents or affected sibling and identified the shared variants. Then, we compared the shared variants in the affected individuals with the ethnic Han Chinese Beijing genomes available in the 1000 Genomes Project (http://www.1000genome.org), and against the Han Chinese Beijing SNPs in the dbSNP131. The changes shared by the affected individuals that were not present in the controls were identified. Sanger sequencing validation and segregation analysis was carried out, and the pathogenic mutations were identified.

### Pathogenic Mutations Identified

In total, 16 pathogenic mutations were identified in 7 of the 17 adRP (41%) families and in 6 of the 17 arRP (35%) families. These included 7 heterozygous, 3 homozygous, and 6 compound heterozygous mutations (Table 1). Of the 16 variants identified, 5 (31%) were reported to be pathogenic, the 11 others (69%) were novel and predicted to be pathogenic. The frequency of the identified genes in the 13 families was, in decreasing order: *RHO* 23% (3/13); *PRPF31*, *CNGA1*, and *USH2A* 15% (2/13); and *PRPF8*, *RDS*, *PDE6A*, and *C2orf71* 8% (1/13). Clinical data of the probands with potential pathogenic mutations are listed in Table 2.

**Table 1:** Mutations identified in the present study.

Family Number	Inheritance	Gene	Genotype	Allele			Note	Computational prediction			Allele frequency		Reference
				Exon	Nucleotide 1	Protein		SIFT	PolyPhen-2	Mutation Taster	controls	ExAC	
RP98	AD	RHO	Heterozygous	Exon 1	c.34delC	p.F13SfsX34	Novel	not tolerated	-	disease causing	0/100	0	
RP154	AD	RHO	Heterozygous	Exon 5	c.1040C>T	p.P347L	reported	-	-	-	-	0.000008263	Dryja TP et al., 1990
RP152	AD	RHO	Heterozygous	Exon 3	c.632A>T	p.H211L	Novel	not tolerated	probably damaging	disease causing	0/100	0	
RP173	AD	PRPF31	Heterozygous	Intron 10	c.1081+19del17bp		Novel	-	-	-	0/100	0	
RP137	AD	PRPF31	Heterozygous	Exon 5	c.393insA	p.N131KfsX22	Novel	-	-	disease causing	0/100	0	
RP128	AD	PRPF8	Heterozygous	Exon 43	c.7008A>G	p.*2336W	Novel	not tolerated	-	disease causing	0/100	0.000008249	
RP214	AD	RDS	Heterozygous	Exon 2	c.589A>G	p.K197E	reported	-	-	-	-	0	Kohl S et al., 1997
RP88	AR	CNGA1	Homozygous	Exon 12	c.1477C>T	p.R493*	Novel	not tolerated	-	disease causing	0/100	0.00001657	
RP135	AR	CNGA1	Homozygous	Exon 5	c.472delC	p.L158FfsX3	Novel	not tolerated	-	disease causing	0/100	0.00009129	
RP352	AR	PDE6A	Homozygous	Exon 10	c.1268delT	p.L423*	Novel	not tolerated	-	disease causing	0/100	0	
RP89	AR	c2orf71	Compound Heterozygous	Exon 1	c.1273C>T	p.R425*	Novel	not tolerated	-	disease causing	0/100	0	
				Exon 1	c.1514G>A	p.W505*	Novel	not tolerated	-	disease causing	0/100	0.000008352	
RP71	AR	USH2A	Compound Heterozygous	Intron 42	c.8559-2A>G		reported	-	-	-	-	0	Dai H et al., 2008
				Exon 57	c.11156G>A	p.R3719H	reported	-	-	-	-	0.00005769	Chen X et al., 2014
RP110	AR	USH2A	Compound Heterozygous	Exon 40	c.7569G>A	p.W2523*	Novel	not tolerated	probably damaging	disease causing	0/100	0	
				Exon 55	c.10859T>G	P.I3620T	reported	-	-	-	-	0.000008238	Katagiri S et al., 2014

**Table 2:** Clinical data are listed for probands from 13 families.

Family Number	Sex	Age (years) at		Visual Acuity		Fundus Features
		Exam	Onset	R eye	L eye	
RP98	M	51	25	0.2	HM	Bilateral retinal vascular attenuation, bone spicule like pigmentation throughout the fundus, chorioretinal degeneration, pale optic disc
RP154	M	38	28	0.5	0.4	Bilateral attenuation of retinal arterioles, bone spicule like pigmentation in the mid-periphery retina, RPE degeneration, pale optic disc
RP152	M	36	24	0.4	0.3	Bilateral attenuation of retinal arterioles, bone spicule like pigmentation in the mid-periphery retina, RPE degeneration, pale optic disc
RP173	M	23	17	0.8	0.8	Bilateral attenuation of retinal arterioles, bone spicule like pigmentation in the periphery retina
RP137	M	43	12	0.4	0.2	Bilateral attenuation of retinal arterioles, bone spicule like pigmentation in the mid-periphery retina, RPE degeneration, pale optic disc
RP128	F	23	3	0.8	0.8	Bilateral attenuation of retinal arterioles, widespread RPE degeneration, macular epimembrane
RP214	F	32	Childhood	0.3	0.4	Bilateral attenuation of retinal arterioles, widespread RPE degeneration, the degenerated RPE area confused in the macular.
RP88	M	48	Childhood	FC	FC	Bilateral retinal vascular attenuation, bone spicule like pigmentation throughout the fundus, chorioretinal degeneration, pale optic disc
RP135	M	36	5	0.3	0.2	Bilateral attenuation of retinal arterioles, bone spicule like pigmentation in the mid-periphery retina, RPE degeneration, pale optic disc
RP352	F	42	Childhood	0.05	0.05	Bilateral retinal vascular attenuation, bone spicule like pigmentation in the mid-periphery retina, RPE degeneration, pale optic disc
RP89	M	45	Childhood	LP	LP	Bilateral retinal vascular attenuation, bone spicule like pigmentation throughout the fundus, chorioretinal degeneration, pale optic disc
RP71	F	37	18	0.6	0.5	Bilateral attenuation of retinal arterioles, bone spicule like pigmentation in the mid-periphery retina
RP110	M	65	25	0.1	0.2	Bilateral retinal vascular attenuation, bone spicule like pigmentation throughout the fundus, RPE degeneration, pale optic disc

FC, finger counting; HM, hand movement; LP, light perception; F, female; M, male.

#### RHO

**Fig 1:**
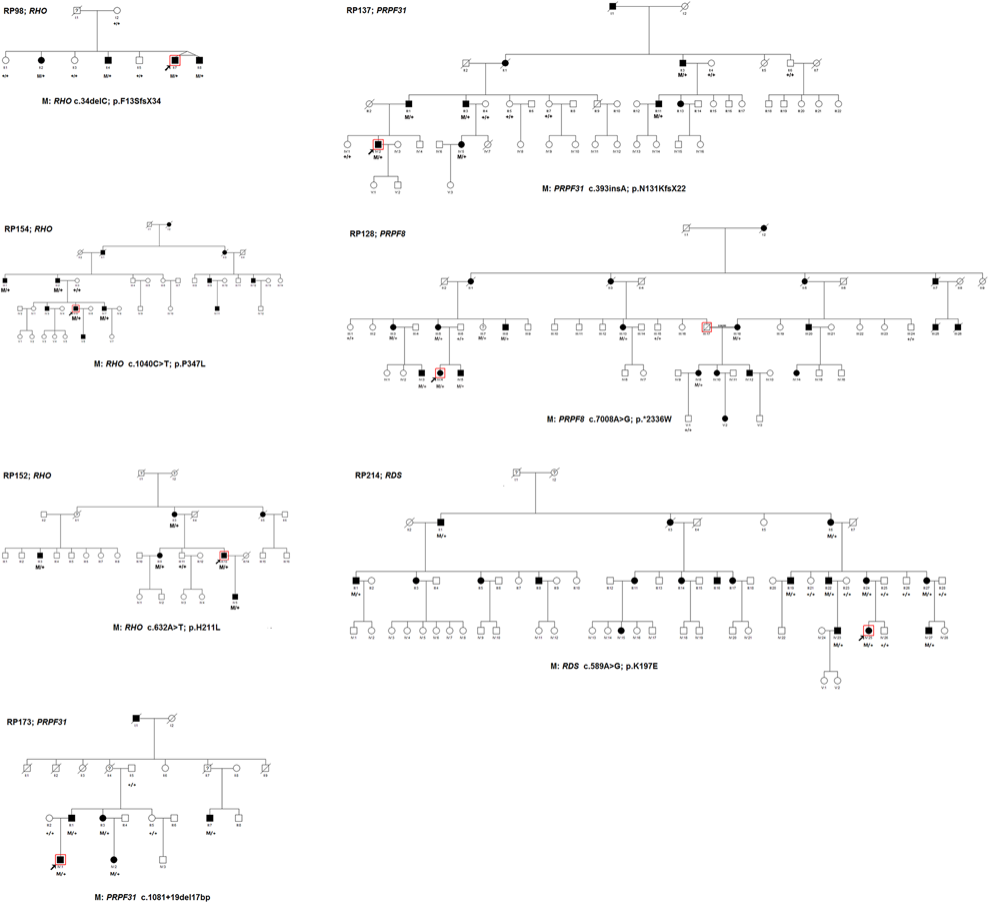
Pedigrees of seven Chinese adRP families and co-segregation analysis in available family members. Filled symbols represent affected, unfilled unaffected. Question marks indicate that it is not clear whether the individual is affected or not. Square signify male, circles females. Arrows mark the index patients. M refers to the mutant allele, and + means normal allele.

**Fig 2:**
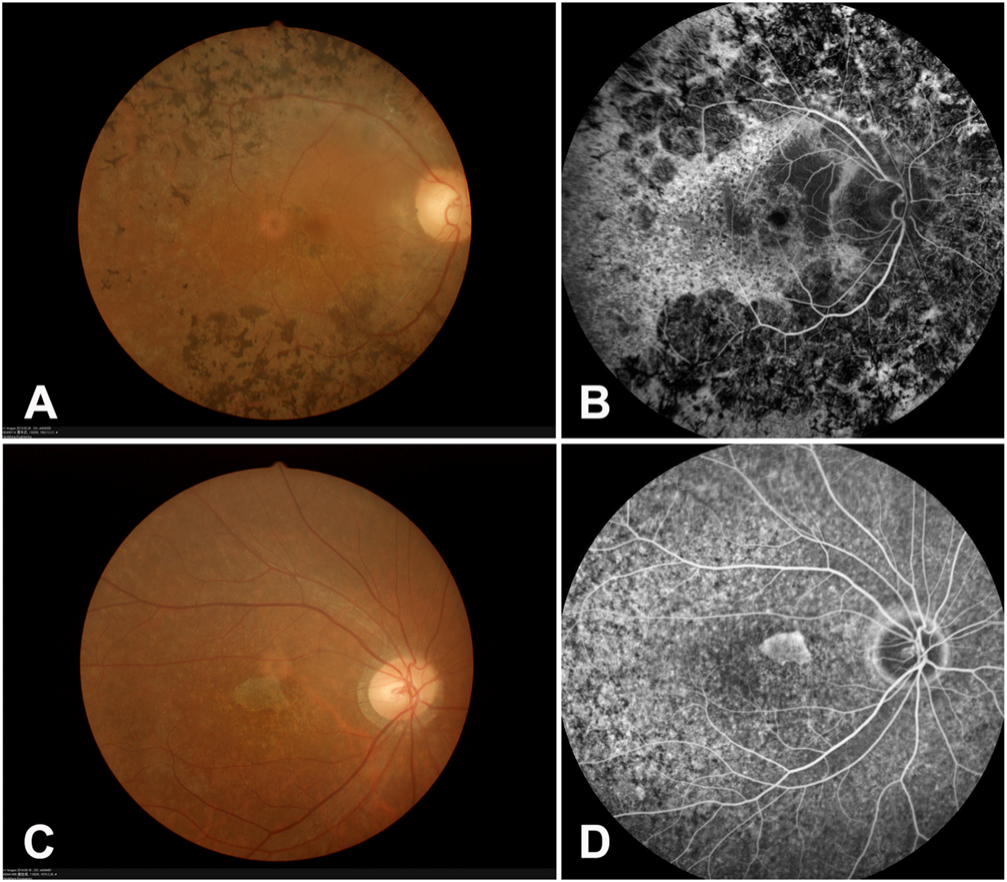
Representative fundus photography and fundus fluorescein angiography (FFA) images from RP98 and RP214 families. (A-B) Proband of RP98. Fundus photography and fluorescein angiography (FFA) showing typical RP changes, including bone spicule-like pigmentation, retinal vascular attenuation, pallor of optic disc and chorioretinal degeneration. (C-D) Proband of RP214. Fundus photography and FFA showing attenuated arterioles and widespread RPE degeneration, the degenerated RPE area confused in the macular.

Three adRP families were found to carry pathogenic mutations in *RHO* (NM_000539). More specifically, the RP98 family carries a heterozygous small deletion c.34delC (p.F13SfsX34), the RP154 family carries a heterozygous missense substitution c.1040C>T (p.P347L), and the RP152 family carries a heterozygous missense substitution c.632A>T (p.H211L). Co-segregation analysis showed that the three mutations co-segregated with the disease phenotype in the tested family members, and was absent in 100 controls (Fig 1). The missense substitution c.1040C>T (p.P347L) was reported as pathogenic and is a hotspot in adRP families [11–13]. The missense substitution c.632A>T (p.H211L) is novel, but the pathogenic variants p.H211P and p.H211R were previously reported [14–15]. The variant p.H211L affected a conserved Histidine, and was predicted to be detrimental using SIFT, PolyPhen-2, and Mutation Taster software. The small deletion c.34delC (p.F13SfsX34) is novel and results in a frameshift leading to 33 new downstream amino acids and premature termination. Fundus examination and fundus fluorescein angiography (FFA) in the 51-year-old proband of the RP98 family showed typical RP changes, including bone spicule-like pigmentation in the retina, retinal vascular attenuation, pallor of the optic disc, and chorioretinal degeneration (Fig 2A and 2B). The proband’s father passed away 48 years ago at 33 years of age. When he died, his visual acuity was relatively normal, and no one in the family can say clearly whether he had night blindness or not. Although it is very likely that he was genetically affected, without evidence, we cannot confirm him as a patient, explaining the original misdiagnosis as autosomal recessive family inheritance.

#### PRPF31

Two adRP families were found to carry two novel variants in *PRPF31* (NM_015629). More specifically, the RP173 family carries a heterozygous small deletion in intron 10 c.1081+19del17bp, and the RP137 family carries a heterozygous small insertion c.393insA (p.N131KfsX22). Co-segregation analysis showed that both mutations co-segregated with the disease phenotype in the tested family members, and was absent in 100 controls (Fig 1). The novel insertion c.393insA (p.N131KfsX22) resulted in a frameshift, which led to premature termination. The small deletion in intron 10 (c.1081+19del17bp) was predicted to be detrimental with AUGUSTUS (http://bioinf.uni-greifswald.de/augustus/submission) and Automated Splice Site Analyses (https://splice.uwo.ca/). As expected based on normal splicing, mRNA RT-PCR analysis of exons 7–12 of *PRPF31* yielded a 590-bp product in control samples. However, no segment was amplified from the affected patients. Without direct sequence evidence, this variant was considered as a possible pathogenic variant. According to previous studies [16], *PRPF31* mutants bearing premature termination codons before the last exon behave as a null allele, resulting in haploinsufficiency as the corresponding mRNA is degraded by nonsense-mediated mRNA decay. Since both mutations result in a premature termination codon before the last exon, haploinsufficiency seems to be the most probable genetic cause of RP in these two families.

#### RDS

The RP214 family was found to carry a reported substitution c.589A>G (p.K197E) in *RDS* (NM_000322). The variant co-segregated with the disease phenotype in the tested family members, and was absent in 100 controls (Fig 1). This substitution was previously reported as pathogenic in a family with cone-rod dystrophy [17]. To rule out the possibility of a misdiagnosis, we clinically reevaluated this family. Most of the affected patients complained of night blindness since childhood and their visual acuity reached 0.8–1.0 during adolescence. At 41 years of age, the visual acuity of IV:23 was 0.7–0.8. As a result, we confirmed that the family indeed has RP, not cone-rod dystrophy. Fundus examination and FFA in the 32-year-old proband revealed attenuated arterioles and widespread RPE degeneration, the degenerated RPE area confused in the macular (Fig 2C and 2D).

#### PRPF8

**Fig 3:**
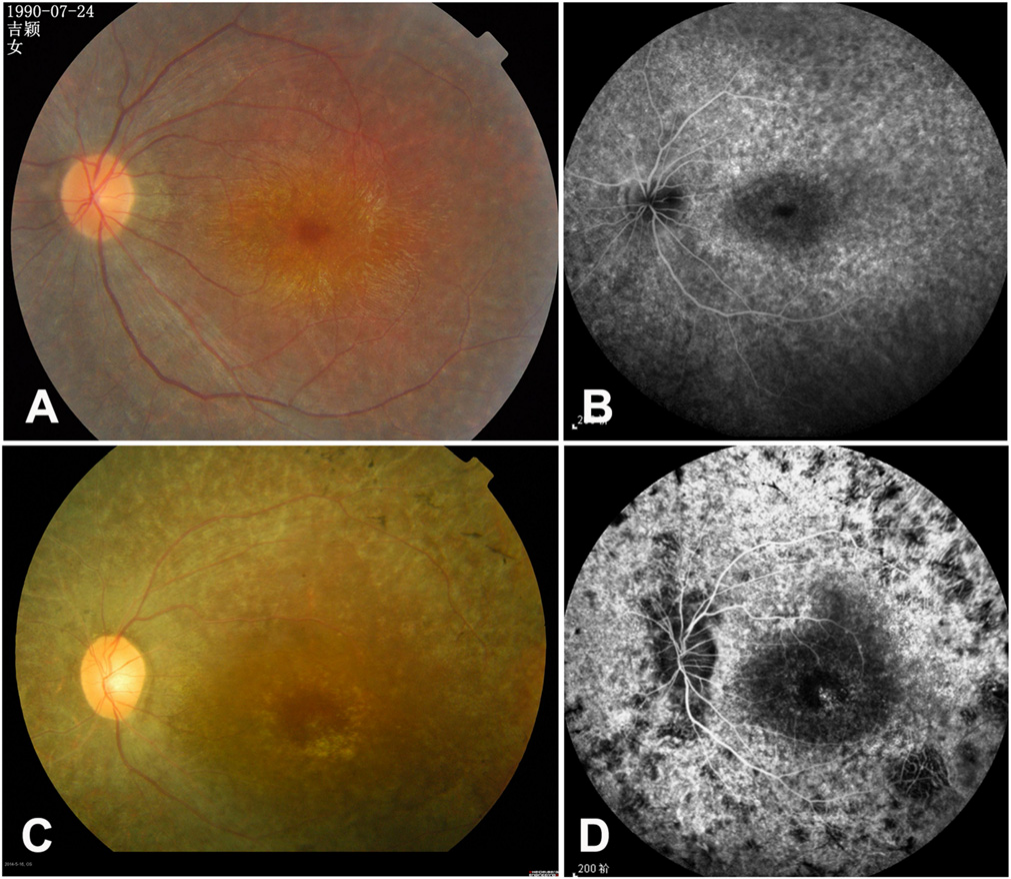
Representative fundus photography and fundus fluorescein angiography (FFA) images from RP128 and RP352 families. (A-B) Proband of RP128. Fundus photography and FFA showing attenuated arterioles, widespread RPE degeneration and macular epimembrane. (C-D) Proband of RP352. Fundus photography and FFA showing typical RP changes, including bone spicule-like pigmentation, retinal vascular attenuation, widespread RPE degeneration and pallor of optic disc.

The RP128 family was found to carry one novel heterozygous mutation c.7008A>G (p.*2336W) in *PRPF8* (NM_006445). The variant was absent in 100 controls (Fig 1). To date, 26 variants in *PRPF8* have been identified to be associated with adRP (Human Gene Mutation Database; http://www.hgmd.cf.ac.uk). The mutation in this study resulted in a longer protein with 41 new downstream amino acids. In this family, all tested affected individuals except III:7 complained of night blindness since childhood. Fundus examination and FFA in the 23-year-old proband showed attenuated arterioles, widespread RPE degeneration, and a macular epimembrane (Fig 3A and 3B). A 56-year-old asymptomatic carrier (III:7) family member did not complain of night blindness, and fundus examination showed no RP changes in both eyes (data not shown), suggesting incomplete penetrance of the *PRPF8* mutation.

#### CNGA1

**Fig 4:**
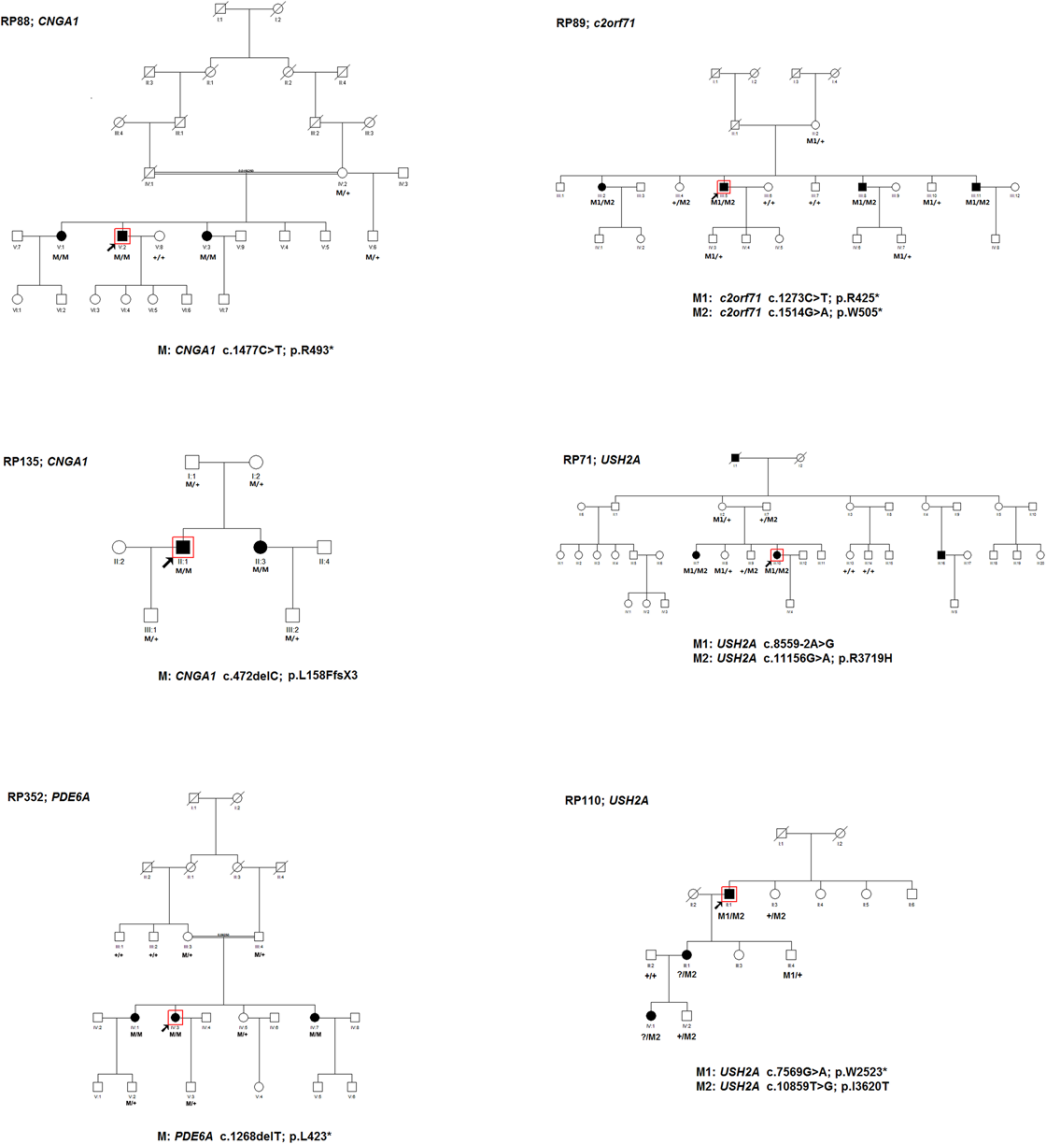
Pedigrees of six Chinese arRP families and co-segregation analysis in available family members. Filled symbols represent affected, unfilled unaffected. Square signify male, circles females. Arrows mark the index patients. The doubled line indicates consanguineous marriage. M refers to the mutant allele, and + means normal allele.

Two arRP families were found to carry novel homozygous mutations in *CNGA1* (NM_001142564). More specifically, the consanguineous RP88 family carries a homozygous nonsense mutation c.1477C>T (p.R493*), and the RP135 family carries a small homozygous deletion c.472delC (p.L158FfsX3). Co-segregation analysis showed that the two mutations co-segregated with the disease phenotype in the tested family members, and was absent in 100 controls (Fig 4). These two variants caused premature termination of exon 12 and exon 5 respectively, resulting in a truncated protein or nonsense-mediated decay. Haploinsufficiency or loss-of-function seems to be the most probable causal mechanism for RP in these two families.

#### PDE6A

The consanguineous RP352 family (which was originally diagnosed with Leber congenital amaurosis at the Tongren Eye Center) was found to carry a novel homozygous small deletion c.1268delT (p.L423*) in *PDE6A* (NM_000440). The variant co-segregated with the disease phenotype in the tested family members, and was absent in 100 controls (Fig 4). The variant resulted in an amino acid change from Lysine to a stop codon (p.L423*) in exon 10. Fundus examination and FFA in the 42-year-old proband showed typical RP changes, including bone spicule-like pigmentation in the mid-periphery retina, bilateral attenuation of retinal vessels, RPE degeneration, and pale optic discs (Fig 3C and 3D). Molecular genetic diagnosis confirmed that the affected individuals in this family have RP, not LCA.

#### C2orf71

The RP89 family carries novel compound heterozygous mutations c.1273C>T (p.R425*) and c.1514G>A (p.W505*) in *C2orf71* (NM_001029883). The variants co-segregated with the disease phenotype in the tested family members, and were absent in 100 controls (Fig 4). Since both variants resulted in premature termination before the last exon, haploinsufficiency seems to be the most probable cause of RP in this family. *C2orf71* (NM_001029883) encodes a 1288-amino-acid photoreceptor-specific protein with unknown function. Its sub-cellular localization is hypothesized to be within the outer segment and/or the connecting cilium of the photoreceptor cells. Its protein sequence motifs suggest that it might be involved in post-translational lipid modification [18].

#### USH2A

Two arRP families were found to carry compound heterozygous mutations in *USH2A* (NM_206933). More specifically, the RP71 family carries compound heterozygous mutations for c.8559-2A>G and c.11156G>A (p.R3719H). The variants co-segregated with the disease phenotype in the tested family members, and were absent in 100 controls (Fig 4). Both variants were first reported in Chinese patients with Usher syndrome type II [19–20], suggesting that these alleles might be pathogenic hotspots in Chinese patients. Compound heterozygous mutations for c.7569G>A (p.W2523*) and c.10859T>G (p.I3620T) were identified in the 65-year-old proband of the RP110 family. The nonsense mutation c.7569G>A (p.W2523*) was novel, and the missense substitution c.10859T>G (p.I3620T) was previously reported in arRP patients [21]. The proband passed the variant p.I3620T to his affected daughter and granddaughter (Fig 4). Based on the autosomal recessive inheritance pattern, there must be another pathogenic variant of *USH2A* in these two patients, however, we only detected several polymorphisms by HEDEP, including c.373G>A (p.A125T), c.7783G>A (p.A2595T), c.9340C>T (p.P3114S), c.10232A>C (p.E3411A), and c.11504C>T (p.T3835I). All polymorphisms were predicted to be benign by SIFT, PolyPhen-2, and Mutation Taster. To clarify the ambiguity between the molecular and clinical diagnosis, the affected members from both families undertook audiometry to exclude the possibility of misdiagnosis. The results showed that the patients had normal hearing sensitivity at 10 dBHL in both ears for most sound frequencies tested. As a result, we confirmed that both families indeed have RP, and not Usher syndrome.

## Discussion

Through targeted exon sequencing and Sanger sequencing-based co-segregation analysis, 16 pathogenic mutations in 8 genes were identified in 13 out of 34 Chinese RP families, achieving a diagnostic rate of approximately 40%. Interestingly, approximately 69% of the pathogenic mutations we identified were novel.

Targeted exon sequencing approaches, which focus on a panel of known candidate genes with deep coverage, allow for the unbiased and accurate identification not only of point mutations and small indels, but also of large exonic deletions and insertions. The results from this study showed a genetic diagnostic rate of approximately 40%, which is significantly higher than that achieved by conventional methods [6–7]. A recent study using a panel of 66 genes reported a diagnostic yield of 82% [22], and some other published RP panels containing more than 100 genes, reported diagnostic yields ranging from 35%-73% in populations from Western European origin [5, 23–26]. Wang J et al. reported that these wide variations were due to differences in data quality, the depth and consistency of coverage, and the percentage of insufficiently covered sequences and uncharacterized regions [22]. Fu Q et al. reported a diagnostic rate of approximately 40% in Chinese arRP patients with a panel of 163 retinal disease genes, and 63% of the identified pathogenic mutations were novel [8]. Xu Y et al. reported a diagnostic rate of approximately 50% in Chinese RP patients based on exome sequencing, and 74% of the identified mutations were novel [27]. In the present study, 69% of the identified pathogenic mutations were novel, which is a higher rate than that observed in similar studies on patients of European descent (45%), suggesting that Chinese individuals have a different genetic background. Targeted re-sequencing is a fast and cost-effective tool for the molecular diagnosis of RP, and can have a practical application in clinics. Nevertheless, the use of a capture system for the enrichment of the target sequences followed by ultra-high throughput sequencing still involves complex technologies that are not standardly available in many laboratories.

Different retinal diseases have many overlapping clinical features, making an accurate clinical diagnosis very challenging, especially in the case of late-stage retinal dystrophy. A comprehensive molecular diagnosis, based on target exon sequencing by screening both known RP genes and other retinal disease genes, can help clinicians to provide a more accurate clinical diagnosis, which can guide better management of the patient and the disease. This study illustrated the value of molecular diagnosis, e.g. for family RP352, which was originally misdiagnosed with Leber congenital amaurosis, while molecular methods confirmed they have RP. In some families with a limited pedigree size, such as family RP110 in our study, it is difficult to determine whether the inheritance pattern is adRP or arRP. In these cases, molecular genetic testing can help to determine the actual mode of inheritance. It is also possible that dominant mutations have incomplete penetrance, causing the parents to not manifest disease phenotypes despite carrying the mutations, as illustrated in family RP128 in this study. Given a transmission risk of 50% for autosomal dominant inheritance, accurate molecular genetic diagnosis can pave the way for better family planning.

It is worth noting that for 21 families in this study, we did not identify defects in the analyzed gene set. Moreover, the other pathogenic mutation of patient III:1 and IV:1 in family RP110 was not identified. In family RP110, the proband’s daughter and grand-daughter have a phenotype that is similar to that of the proband, suggesting that they might also carry a compound heterozygous mutation in the same gene as the proband. This leaves the possibility that some of the unsolved families may harbor pathogenic mutations in known genes. There are five possible reasons for not finding these mutations by targeted exon sequencing: (1) the sequence coverage in the targeted exon sequencing was low in some areas; (2) there are larger deletions or rearrangements not detectable by exon sequencing; (3) there are deeper intronic mutations causing aberrant splicing as shown for other genes; (4) there are mutations in regulatory regions not targeted by our study; or (5) mutations in a gene not currently associated with RP are responsible for the disease [28]. For those patients whose DNA analysis does not yield a molecular diagnosis by this panel, careful re-assessment of clinical and family history, including possible examination of extended family members, may be considered.

In summary, targeted capture sequence approaches offer a rapid, effective, and accurate method for the molecular diagnosis of RP, which helps to provide a more accurate clinical diagnosis and subsequently paves the way for family planning in affected families.

## Supporting Information

S1 Fig Representative sequence chromatograms for seven adRP families. (TIF)

S2 Fig Representative sequence chromatograms for three consanguineous arRP families. (TIF)

S3 Fig Representative sequence chromatograms for three arRP families which carried compound heterozygous mutations. (TIF)

S1 Text Some of the Raw data can be downloaded at the website http://pan.baidu.com/share/link?shareid=1352816587&uk=2132102424. (XLSX)
