# Supplementary figures and images for: Dependable and Efficient Clinical Molecular Diagnosis of Chinese RP Patient with Targeted Exon Sequencing
Source: PLoS One. 2015 Oct 23;10(10):e0140684. doi: 10.1371/journal.pone.0140684 (PMC4619688; doi:10.1371/journal.pone.0140684)

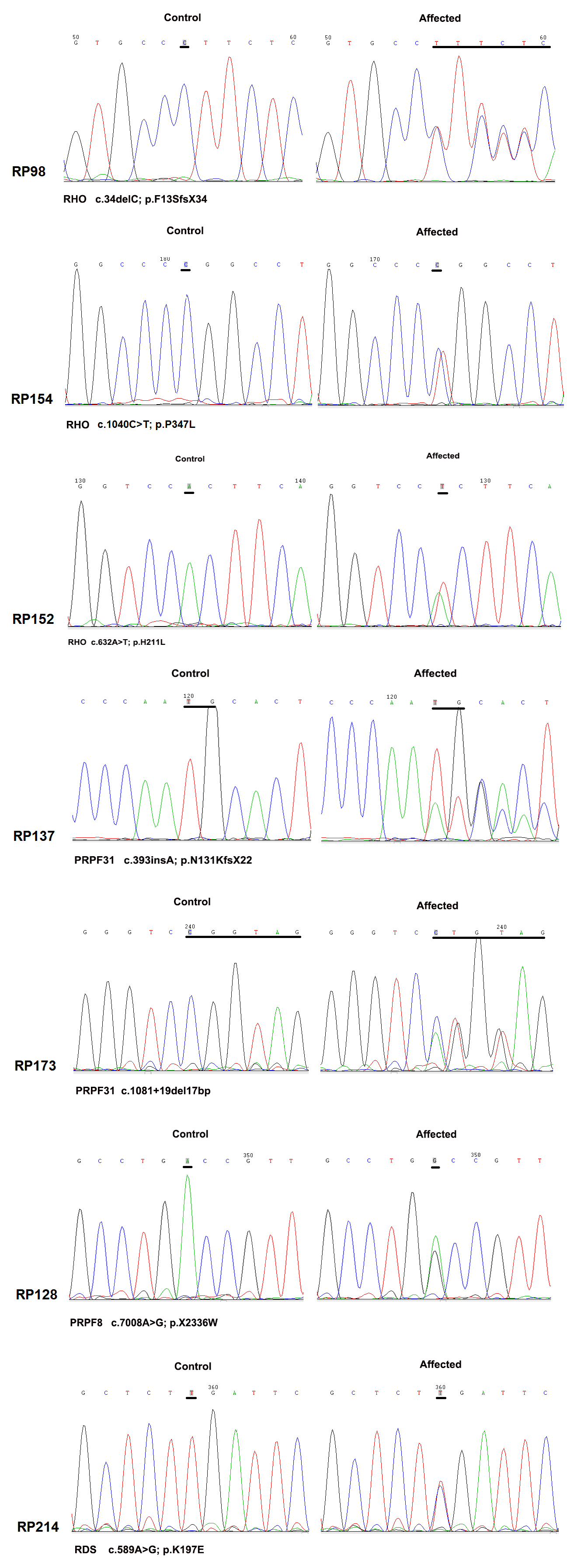

Supplement: S1 Fig — (TIF) [file pone.0140684.s001.tif]

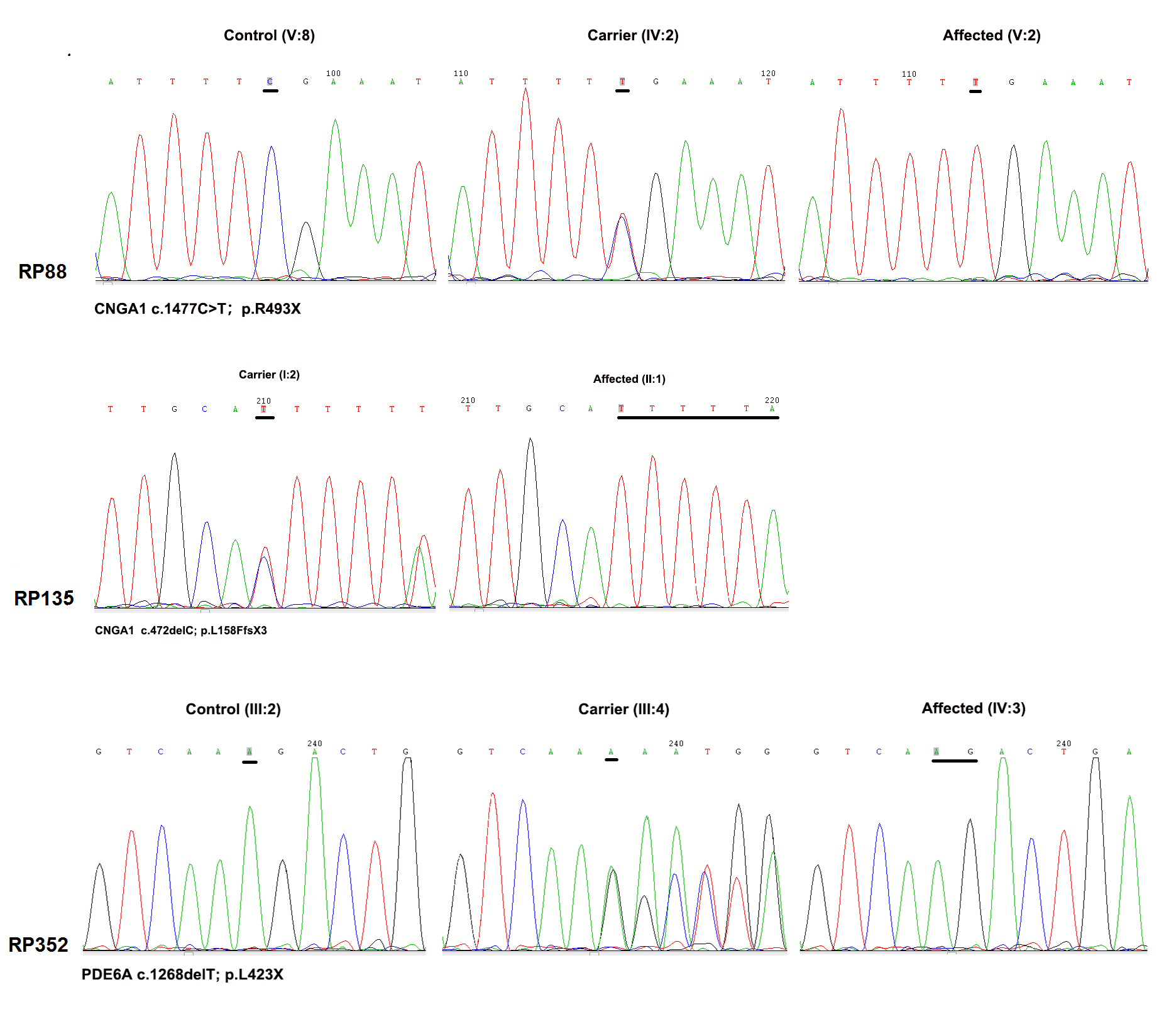

Supplement: S2 Fig — (TIF) [file pone.0140684.s002.tif]

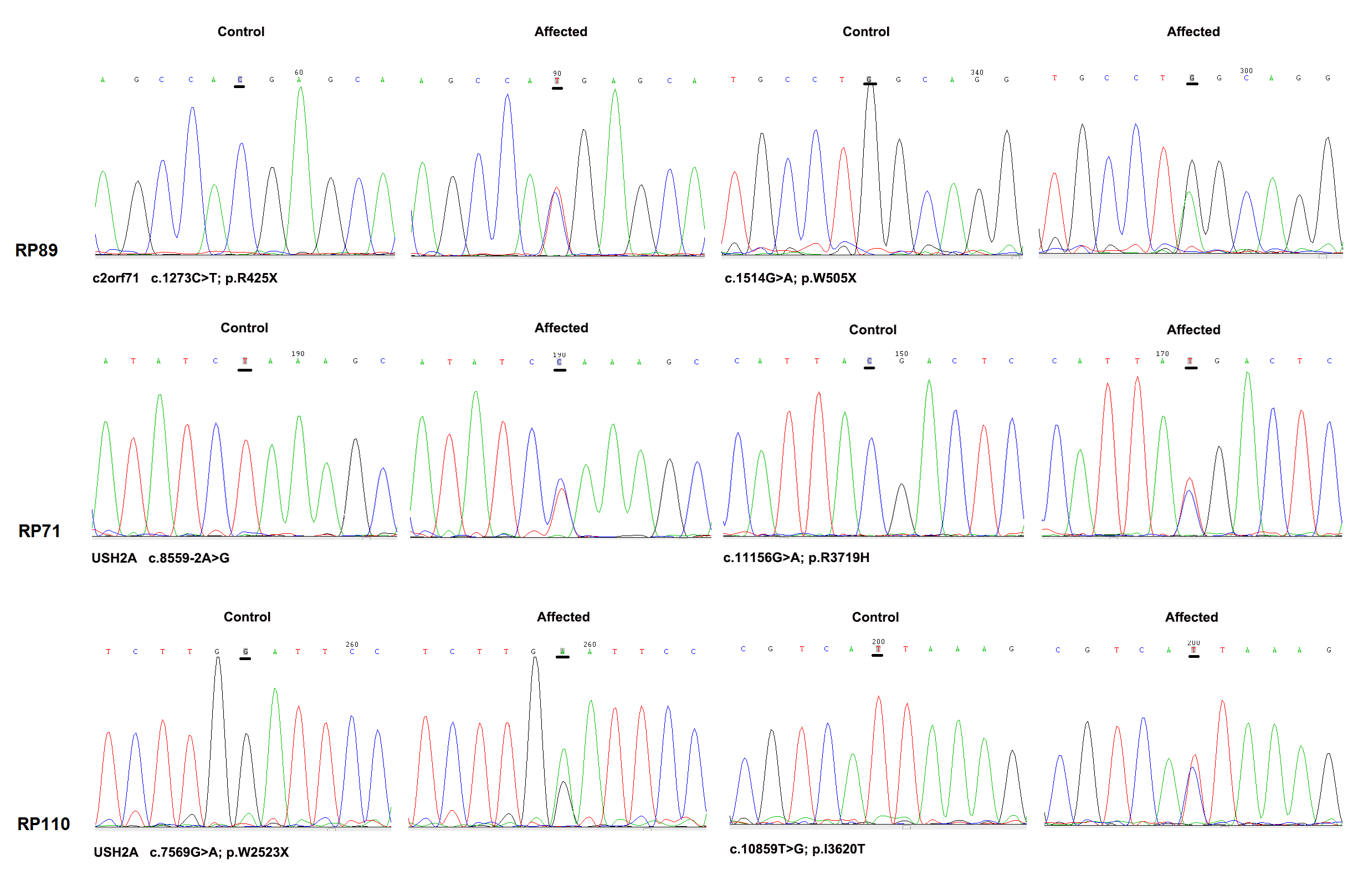

Supplement: S3 Fig — (TIF) [file pone.0140684.s003.tif]
